# Relationship between Metabolic Syndrome and Lower Urinary Tract Symptoms: Hallym Aging Study

**DOI:** 10.1155/2015/130917

**Published:** 2015-06-23

**Authors:** Seong Ho Lee, Sang Kon Lee, Min Soo Choo, Kyung Tae Ko, Tae Young Shin, Won Ki Lee, Tsolmon Batsaikhan, ShanAi Quan, Jin Young Jeong, Dong Hyun Kim

**Affiliations:** ^1^Department of Urology, School of Medicine, Hallym University, 1153 Kyo-dong, Chuncheon, Gangwon 200-704, Republic of Korea; ^2^Department of Child and Adolescent Health, College of Public Health, Zhengzhou University, 100 Kexue Road, Zhengzhou 450001, China; ^3^Department of Social and Preventive Medicine, School of Medicine, Hallym University, Chuncheon, Gangwon 200-702, Republic of Korea; ^4^Hallym Research Institute of Clinical Epidemiology, Hallym University, Chuncheon, Gangwon 200-702, Republic of Korea

## Abstract

The aim of the study was to test the hypothesis that the metabolic syndrome (MS) is linked to lower urinary tract symptoms (LUTS) in Korean men. This was a longitudinal study that used data collected from 328 men aged 50–89 years who were randomly selected among 1,520 participants in 2004. We collected information from 224 (68.3%) men among the original responders on the biological, medical, psychological, social, lifestyle, and economic factors in 2007. The prevalence of the MS was 187/328 (57.0%) in 2004 and 125/224 (55.8%) in 2007 among men, respectively. There was no significantly greater increase in the IPSS in men with the MS than in men without the MS over a 3-year period of time (2.0 ± 9.37 versus 3.0 ± 8.44, *p* = 0.402, resp.). In the multivariate logistic regression analysis with control for age and life style factors, the risk factors for moderate/severe LUTS were age and erectile dysfunction (*p* < 0.05). However, the presence of the MS did not increase the risk of moderate/severe LUTS (OR = 1.09, 95% CI 0.63–1.89, *p* = 0.748). Our cross-sectional and longitudinal risk factor analyses do not support the hypothesis that the MS is linked to LUTS in Korean men.

## 1. Introduction

Lower urinary tract symptoms (LUTS) are highly prevalent and are a chronic progressive condition in older men. An age-related increase in symptom scores is evident, increasing by about 0.8 per year [1].

Several epidemiological studies assessing the association between LUTS and diabetes suggest that diabetes is associated with an increased LUTS severity [2–4]. LUTS have been associated with components of the metabolic syndrome (MS) such as hypertension, fasting blood sugar (FBS), erectile dysfunction (ED), and lifestyle factors [5–10]. MS is associated with an increased risk of cardiovascular disease and diabetes mellitus. The main components of the MS are obesity and elevated fasting plasma glucose level [11]. The prevalence of the MS increases with age [12]. Estimated prevalence of the MS in the USA, as defined by the Adult Treatment Panel (ATP) III report, increased from 6.7% among participants aged 20–29 years to 43.5% and 42% among participants aged 60 through 69 years and among those aged at least 70 years, respectively [12]. Recently, the prevalence of type 2 diabetes has rapidly increased in native and migrant Asian populations. Diabetes develops at a younger age than in white populations [13]. In the WHO-defined obesity group, the obesity prevalence was increased by 11% from 26.8% to 37.8% during 1998–2012 in Korean men above 30 years of age [14]. The prevalence of hypertension and diabetes was increased by 12% from 47.3% to 59.3% and by 6.2% from 18.1% to 24.3% in men above 65 years of age, respectively. The prevalence of the MS using the ATP III criteria was 6% for Japanese and 12% for Mongolians, a remarkably lower prevalence relative to the reported prevalence in the USA [15].

However, the role of the MS in the etiology of LUTS in elderly men is not clear. The results of investigation on the MS as a risk factor for LUTS are inconsistent. Several studies, but not all, have reported the association between MS and LUTS [16–21]. In the Boston Area Community Health Survey, the prevalence of the MS was increased to 40% with mild LUTS (AUA-SI of 2–7), but interestingly it stabilized with moderate/severe symptoms [4]. The results indicated other underlying factors affecting the severity of LUTS. In a health survey in Japan and a large Chinese population-based investigation, the MS was not significantly associated with the severity of LUTS [18, 21]. The aim of the study was to test the hypothesis that MS is associated with LUTS in middle aged and elderly men living in the suburban city, Chuncheon.

## 2. Materials and Methods

### 2.1. Study Population

The Hallym Aging Study (HAS) is a prospective cohort study, which was initiated to investigate qualities of life and health among elderly community residents in Chuncheon city, located about 120 km east of Seoul. This ongoing study commenced in 2004 and involves follow-up examinations at 3 yr intervals. The eligibility criteria applied were an age of more than 50 yr, residence within the borders of the survey area for at least 6 months before testing, and the mental and physical ability to participate. Two hundred of 1,408 census tracts were randomly sampled to represent residential areas proportionately based on the Korean National Census conducted in 2000. In addition, study subjects were selected so that those over 65 years of age represent about 70% of the study cohort. During the 2004 survey, 918 subjects were interviewed among 1,510 eligible subjects (response rate 61%). The present study involved 702 subjects that participated in the 2007 follow-up survey (64 subjects had died, 49 had moved, and 103 refused to participate or could not be contacted).

### 2.2. Data Collection and Clinical Measurements

Sociodemographic characteristics, such as age, education level, and marital status, and behavioral characteristics, such as drinking, smoking, and regular exercise, and other data, such as past medical history, depression, and erectile dysfunction, were collected through face to face interviews by trained interviewers.

Clinical tests including anthropometry, blood pressure measurement, and blood test were performed by the hospital clinical teams (Departments of Internal Medicine, Family Medicine, Urology, Laboratory Medicine, and Diagnostic Radiology, Chuncheon Sacred Heart Hospital, Hallym University). Height and weight were recorded using an automatic measurement system (DS-102, JENIX, Seoul, Republic of Korea), and body mass index (BMI) was calculated using the following formula: [weight (kg)/height (m^2^)].

For blood pressure, after maintaining a stable state for over 10 min, a skillful resident in family medicine measured each of the systolic blood pressure and diastolic blood pressure twice and used the mean values. For the blood test, blood was sampled early in the morning after more than 10 hrs of fasting and blood sugar was analyzed using Hitachi 747 autoanalyzer (Hitachi, Tokyo, Japan).

### 2.3. The Assessment of LUTS

In all the study participants, LUTS were assessed using a validated questionnaire, the International Prostate Symptom Score (IPSS), and were classified into three categories according to the IPSS: mild (IPSS: ≤7), moderate (IPSS: 8–19), and severe (IPSS: ≥20) [22].

### 2.4. The Assessment of Erectile Function

In all the study participants, erectile function was assessed using a validated questionnaire, the 5-item version of the International Index of Erectile Function (IIEF-5), and was classified into four categories according to the IIEF-5: no ED (IIEF-5: ≥18), mild ED (IIEF-5: 14–17), moderate ED (IIEF-5: 10–13), and severe ED (IIEF-5: ≤9) [23].

### 2.5. The Assessment of the MS

According to the National Cholesterol Education Program Adult Treatment Panel III (NCEP-ATP III) conventional definition [24], MS syndrome was defined if the subjects had more than three of the following five criteria: (1) visceral obesity according to the circumference criteria ≥90 cm or BMI ≥25, (2) high blood pressure ≥130/85 mmHg, (3) high level of fasting glucose, ≥110 mg/dL, (4) low HDL-C <45 mg/dL, and (5) hypertriglyceridemia, ≥150 mg/dL. The definition of obesity was based on modification of the Western Pacific Regional Office of WHO for defining overweight and obesity for Asians [25].

### 2.6. Statistical Analysis

Statistical analysis was performed using SPSS 15.0 for Windows. Comparisons of general characteristics of the study population between groups stratified by LUTS severity were made by using the ANOVA test and the chi-square test. Between the age groups, comparisons were made for the severity of LUTS and the presence of MS using the Mantel-Haenszel chi-square test. The multivariate logistic regression analysis was used to estimate the effect of MS on LUTS. The changes in the MS and LUTS with aging were assessed by using the chi-square test. Changes in the severity of LUTS between groups with and without the MS were compared by using the paired *t*-test and the two-way ANOVA test. A value of *p* < 0.05 was considered statistically significant.

## 3. Results

### 3.1. Characteristics of the Study Population according to the Severity of LUTS

A total of 328 men in the 2004 population were included in this study. The mean age of participants was 69.7 ± 7.8 years, and 36 (11.0%) men were aged 50–59 years, 110 (33.5%) men were aged 60–69 years, 147 (44.8%) men were aged 70–79 years, and 35 (10.7%) men were aged 80 years or older. Of 328 men, 187 (57.0%) had the MS and 135 (41.2%), 130 (39.6%), and 63 (19.2%) men had no/mild, moderate, and severe LUTS, respectively. IIEF-5 increased significantly as the severity of LUTS increased (ANOVA test, *p* < 0.001); however, there was no significant difference in the prevalence of the MS and lifestyle factors such as smoking and regular exercise between groups when stratified by LUTS severity (chi-square test, *p* > 0.05) (Table 1).

### 3.2. Prevalence/Severity of MS and LUTS

**Figure 1:**
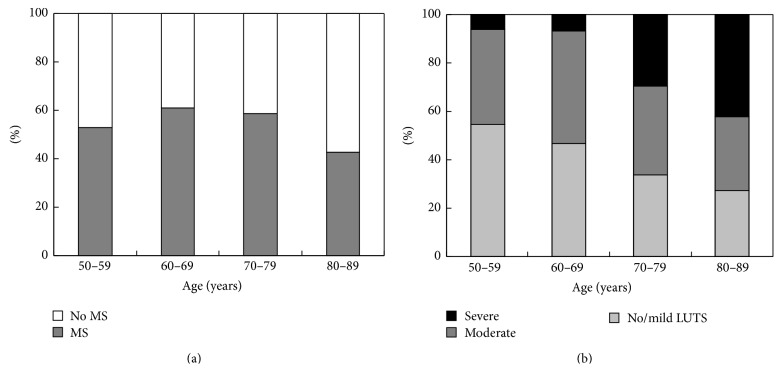
Prevalence and severity of the metabolic syndrome (a) and lower urinary tract symptoms (LUTS) (b) in different age groups of study population.

The number of men with MS was 19 (52.8%), 67 (60.9%), 86 (58.5%), and 15 (42.9%), respectively, among those aged 50–59 years, 60–69 years, 70–79 years, and 80 years or older, and there was no significant difference in the prevalence of MS between the age groups (Mantel-Haenszel chi-square test, *p* = 0.268) (Figure 1). The prevalence and severity of LUTS increased statistically significantly with age (Mantel-Haenszel chi-square test, *p* < 0.005) (Figure 1).

### 3.3. The Relative Risk of Moderate/Severe LUTS according to the Presence of MS

After adjusting for age and lifestyle factors that were related to LUTS in the multiple logistic regression analysis, MS was not found to be associated with the presence of moderate/severe LUTS (OR = 1.09, 95% CI 0.63–1.89, *p* = 0.748) (Table 2).

### 3.4. The Changes in the MS and LUTS with Aging

Of 328 participants in the 2004 study population, 104 (31.7%) men failed to participate in the 2007 follow-up survey and 224 men completed the study in 2007. The mean age of participants in 2007 study was 71.7 ± 7.4 years, and 27 (12.0%) men were aged 50–59 years, 68 (30.4%) men were aged 60–69 years, 92 (41.1%) men were aged 70–79 years, and 37 (16.5%) men were aged 80 years or older. As a number of participants were excluded in the 2007 study, in order to test the effect of the resultant selection bias, we compared the 224 participants included and the 104 excluded and confirmed that the two groups were homogeneous (Table 3). Thus, the effect of selection bias resulting from the exclusion of participants is considered not significant.

Among the 2007 study population, 100 (44.6%), 84 (37.5%), and 40 (17.9%) men had no/mild, moderate, and severe LUTS in 2004, respectively, and 71 (31.7%), 92 (41.1%), and 61 (27.2%) men had no/mild, moderate, and severe LUTS in 2007, respectively. The severity of LUTS increased significantly with aging (*p* = 0.008) (Table 4).

Among the 2007 population, 125 (55.8%) men and 131 (58.5%) men had the MS in 2004 and 2007, respectively, and there was no significant difference in the rate of the MS between the 2004 and 2007 populations (Table 4) (*p* = 0.532).

### 3.5. The Change in the Severity of LUTS according to the Presence of the MS with Aging

Among the 2007 study participants, 99 men did not have the MS in 2004. In these men, storage IPSS, voiding IPSS, and total IPSS were 4.22 ± 4.02, 6.93 ± 6.33, and 11.15 ± 9.34 in 2004 and 5.51 ± 3.86, 7.65 ± 6.00, and 13.16 ± 9.33 in 2007, respectively.

Among the 2007 population, 125 men had the MS in 2004. In these men, storage IPSS, voiding IPSS, and total IPSS were 4.20 ± 3.85, 6.19 ± 6.13, and 10.39 ± 8.54 in 2004 and 5.49 ± 4.01, 7.90 ± 5.62, and 13.4 ± 8.98 in 2007, respectively.

There was no significant difference in the change of storage IPSS, voiding IPSS, and total IPSS between men with and without the MS in 2004 with aging (Table 5).

## 4. Discussion

The most important finding of the present study is that we found no evidence of a link between the MS and LUTS. We have tested this hypothesis in two different ways, using a cross-sectional and a longitudinal risk factor analysis. Both analyses ended up in the same conclusion.

Our results suggest that the difference in prevalence of the MS is not significant between men with mild and moderate-to-severe LUTS. LUTS is significantly associated with aging and IIEF-5 regardless of the presence of MS. However, no association was found between LUTS and regular physical activity, which has been suggested as one of the factors that potentially decrease the risk of LUTS. We previously reported that the risk factors for vascular disease including hypertension, DM, hyperlipidemia, and smoking are significantly associated with LUTS, using data from the same cohort in the Hallym Aging Study [26].

There are several studies that investigated the relationship between the MS and LUTS; however, the results of these studies were inconsistent.

Rohrmann et al. [8] in a large population-based survey found that the components of the MS were likely to be associated with LUTS in older men. However, Temml et al. [16] reported that the association between the MS and LUTS was not evident in both sexes aged 30–69 years, a relatively younger age group compared with those in this study. Park et al. [27] reported that no significant differences were found in the mean IPSS or quality-of-life score between men with or without the MS. Recently, Hong et al. [17] reported that there were no significant differences in LUTS depending on the presence or absence of the MS in males, whereas there were significant differences in LUTS among females.

The possible pathophysiologic mechanism of an association between the MS and LUTS may be related to an increase in the sympathetic activity and IGF [28]. Rats with induced diabetes showed that a decrease in nitric oxide activity may be involved in nitrergic nerve degeneration [29]. Autonomic hyperactivity, a component of the MS, increases the sympathetic tone [30]. Rapidly developing BPH was associated with diseases linked to sympathetic overactivity, including obesity, type 2 diabetes mellitus, and hypertension [31].

The BACH survey showed that the increased prevalence of the MS was associated with LUTS although mild, primarily in men less than 60 years of age [4]. The prevalence of the MS increases with increasing IPSS in the mild symptom range and stabilizes with moderate-to-severe symptoms. In our study, the prevalence of the MS was 57%, a relatively high rate compared with other Korean survey data because of the difference in the age group of participants.

The prevalence of the MS is variable according to the definition of the MS [18, 32]. In a Korean survey of MS, the age-adjusted prevalence of MS based on the ATP III criteria was 30.1% in men and 37.9% in women, whereas the age-adjusted prevalence of MS based on the Asia-Pacific criteria was 48.2% in men and 46.6% in women, respectively. The prevalence of the MS was increased in the 65–74 age group, and then it gradually decreased in men whereas it increased with age in women [32]. In a Chinese large population-based survey, the prevalence of the MS was markedly increased with age, from 9.6% (age group of less than 40 years) to 23.4% (age group of 40–59 years), and then it reached a plateau [21]. In the longitudinal observation of a large Japanese cohort and a general health checkup survey, the highest prevalence of the MS was observed in men aged 50–64 years [18, 33]. These results suggest that the prevalence of the MS is not dependent on age in the old age group.

There is a well-known correlation between risk factors for vascular disease and LUTS-linked BPH [26, 34, 35].

Visceral obesity has been proposed as the most important determinant of the MS [29]. Recently, the prevalence of obesity is rapidly increasing in Asian countries. In the WHO-defined overweight group, the all-cause mortality risk was significantly increased at BMI ≥25 kg/m^2^ rather than at BMI ≥30 kg/m^2^ in Asians [36]. The study proposed the use of BMI with a new cut-off point for obesity in Asians. According to the 2012 Korea Health Statistics, the prevalence of obesity based on the BMI ≥25 kg/m^2^ criteria was increased during the 5th decade [14]. However, in the previously reported Hallym Aging Study, the highest prevalence rate of obesity was observed in men aged 60–74 years living in the subrural Chuncheon city area [32]. The prevalence of the MS in men increased to that in the similar age group of 55–64 years and then it gradually decreased. This result indicates the role of obesity in the genesis of the MS.

Vascular diseases are presumed to be one of the causes that are responsible for the pathogenesis of LUTS [37]. In an experimental study in rats, pelvic arterial occlusive disease caused progressive vascular damage, resulting in bladder dysfunction [38].

As described earlier, there are several studies reporting the association between MS and LUTS. But most of these studies used a cross-sectional design based on the statistical data collected at a certain time point. These cross-sectional studies are not sufficient to clarify whether the MS induces LUTS or whether two different conditions simply occur as a result of the common etiology. That is, a causal relationship between the two conditions remains obscure.

The HAS is limited geographically to the suburban Chuncheon city area, which may not be fully representative of the Korean population. However, to date, none of the studies have investigated the time-dependent correlations between the two conditions in a single cohort; this is the first study to do so. One of the limitations of this study is that the number of participants is small and the study period is relatively short. A further longer study with sufficient number of participants would demonstrate the link between the MS and LUTS more clearly.

Another limitation is the high dropout rate of study participants. This cohort included 88% of subjects aged 60 years or more who were living in the suburban city. The high proportion of elderly participants and poor accessibility from the suburban area could be the possible explanations for the high dropout rate. Although Gades et al. [39] reported that dropout was not related to primary study outcomes in a twelve-year community-based, prospective cohort study of urologic disease in men and there was no significant difference in several parameters between included and excluded participants in the 2007 study, the high dropout rate could be the risk of bias.

## 5. Conclusions

Our cross-sectional and longitudinal risk factor analyses do not support the hypothesis that the MS is linked to LUTS in Korean men.

## Figures and Tables

**Table 1:** General characteristics for the study population according to severity of LUTS.

	LUTS			Total	*p* value
	No/mild	Moderate	Severe		
Number of respondents (%)	135 (41.2)	130 (39.6)	63 (19.2)	328 (100)	
Age (years)	67.5 ± 8.4	70.9 ± 7.1	71.8 ± 6.5	69.7 ± 7.8	<0.001^a^
MS (%)					
(+)	78 (41.7)	73 (39)	36 (19.3)	187 (100)	0.958^b^
(−)	57 (40.4)	57 (40.4)	27 (19.2)	141 (100)	
WC (cm)	89.0 ± 7.6	87.2 ± 8.2	87.4 ± 9.2	88.0 ± 8.2	0.222^a^
BMI (kg/m^2^)	25.2 ± 2.7	24.5 ± 2.7	24.3 ± 3.5	24.8 ± 2.9	0.085^a^
Hypertension (%)	40 (29.6)	40 (30.8)	23 (36.5)	103 (29.3)	0.612^b^
DM (%)	19 (14.1)	14 (10.8)	10 (15.9)	43 (12.3)	0.561^b^
Hyperlipidemia (%)	7 (5.2)	2 (1.5)	1 (1.6)	10 (2.8)	0.170^b^
IIEF-5	14.5 ± 8.3	10.2 ± 8.9	8.0 ± 8.0	11.5 ± 8.8	<0.001^a^
Height (cm)	162.7 ± 6.9	162.9 ± 6.6	163.2 ± 6.3	162.9 ± 6.6	0.898^a^
Weight (kg)	66.8 ± 9.2	65.4 ± 9.3	64.9 ± 10.4	65.9 ± 9.5	0.351^a^
Total cholesterol (mg/dL)	192.1 ± 35.1	185.8 ± 33.6	189.3 ± 36.5	189.1 ± 34.8	0.347^a^
Education (yr)	7.1 ± 4.6	7.0 ± 4.7	7.5 ± 4.6	7.1 ± 4.6	0.823^a^
Income (10,000 won, %)					0.009^b^
<600	38 (28.1)	67 (51.5)	31 (49.2)	136 (41.5)	
600–1800	44 (32.6)	26 (20.0)	15 (23.8)	85 (25.9)	
1800–3600	22 (16.3)	15 (11.5)	9 (14.3)	46 (14.0)	
>3600	6 (4.4)	2 (1.5)	0 (0)	8 (2.4)	
Smoking (%)					0.883^b^
None	29 (21.5)	24 (18.5)	10 (15.9)	63 (19.2)	
Ex	66 (48.9)	64 (49.2)	31 (49.2)	161 (49.1)	
Current	40 (29.6)	42 (32.3)	22 (34.9)	104 (31.7)	
Regular exercise (%)	41 (30.4)	41 (31.5)	15 (23.8)	73 (22.3)	0.470^b^
Lab					
Total cholesterol (mg/dL)	192.1 ± 35.1	185.8 ± 33.6	189.3 ± 36.5	189.1 ± 34.8	0.347^a^
Uric acid (mg/dL)	5.7 ± 1.5	5.7 ± 1.4	5.5 ± 1.6	5.7 ± 1.5	0.564^a^
Creatinine (mg/dL)	1.02 ± 0.23	1.01 ± 0.19	1.21 ± 1.34	1.05 ± 0.62	0.080^a^
CRP (mg/L)	0.27 ± 0.78	0.33 ± 0.57	0.17 ± 0.17	0.27 ± 0.62	0.331^a^
MMSE	26.5 ± 3.1	25.9 ± 2.8	26.1 ± 2.8	26.2 ± 3.0	0.480^a^
GDS	9.8 ± 6.4	13.8 ± 6.7	15.5 ± 6.5	12.4 ± 6.9	<0.0001^a^

LUTS: lower urinary tract symptoms; MS: metabolic syndrome; WC: waist circumference; BMI: body mass index; DM: diabetes mellitus; IIEF-5: 5-item version of the International Index of Erectile Function; CRP: c-reactive protein; MMSE: Mini-Mental State Examination; GDS: geriatric depression scale. ^a^ANOVA test. ^b^Chi-square test.

**Table 2:** Multivariate logistic regression analysis of risk factors associated with LUTS.

	Moderate-to-severe LUTS Age-adjusted odds ratio (95% confidence interval)	*p* value	Moderate-to-severe LUTS Odds ratio (95% confidence interval)	*p* value
MS				
No	1		1	
Yes	1.005 (0.635–1.589)	0.913	1.094 (0.634–1.886)	0.748
Age	—		1.066 (1.023–1.111)	0.003
IIEF-5	0.960 (0.929–0.992)	0.014	0.956 (0.924–0.989)	0.010
Smoking	1.002 (0.999–1.004)	0.150	1.001 (0.999–1.004)	0.264
Alcohol intake	1.000 (0.998–1.002)	0.994	1.000 (0.998–1.001)	0.774
No regular exercise	1.116 (0.649–1.918)	0.691	1.331 (0.717–2.471)	0.364
Total cholesterol	0.997 (0.990–1.004)	0.304	0.997 (0.989–1.005)	0.471

**Table 3:** Comparisons between included and excluded participants in the 2007 study.

	Included participants (*N* = 224)	Excluded participants (*N* = 104)	*p* value
Age (years)	71.4 ± 7.4	72.8 ± 8.3	0.272
MS (%)	125 (55.8)	61 (58.6)	0.552
WC (cm)	88.3 ± 7.4	87.4 ± 9.4	0.387
BMI (kg/m^2^)	24.92.7	24.6 ± 3.2	0.435
Hypertension (%)	72 (32.1)	32 (30.7)	0.646
DM (%)	28 (12.5)	15 (14.4)	0.650
Hyperlipidemia (%)	9 (4.0)	2 (1.9)	0.217
IIEF-5	12.1 ± 8.7	10.5 ± 9.0	0.150
Smoking (%)			0.867
None	42 (20.9)	21 (20.1)	
Ex	108 (53.7)	53 (50.9)	
Current	51 (25.4)	30 (28.8)	
Regular exercise (%)	67 (33.3)	30 (23.6)	0.134
Alcohol (g/day)	53.2 ± 158.2	39.9 ± 100.0	0.395
Total cholesterol (mg/dL)	190.4 ± 35.2	187.0 ± 34.1	0.403

MS: metabolic syndrome; WC: waist circumference; BMI: body mass index; DM: diabetes mellitus; IIEF-5: 5-item version of the International Index of Erectile Function.

**Table 4:** The longitudinal changes in the metabolic syndrome and LUTS in a total of 224 subjects.

Year	LUTS (%)			MS (%)
	No/mild	Moderate	Severe	
2004	100 (44.6)	84 (37.5)	40 (17.9)	125 (55.8)
2007	71 (31.7)	92 (41.1)	61 (27.2)	131 (58.5)

*p* value	0.008			0.532

**Table:** (a) Storage IPSS

	2004	2007	*p* value^*∗*^
MS (−)	4.22 ± 4.02	5.51 ± 3.86	0.023
MS (+)	4.20 ± 3.85	5.49 ± 4.01	0.010

*p* value^†^	0.984		

**Table:** (b) Voiding IPSS

	2004	2007	*p* value^*∗*^
MS (−)	6.93 ± 6.33	7.65 ± 6.00	0.412
MS (+)	6.19 ± 6.13	7.90 ± 5.62	0.022

*p* value^†^	0.250		

**Table:** (c) Total IPSS

	2004	2007	*p* value^*∗*^
MS (−)	11.15 ± 9.34	13.16 ± 9.33	0.133
MS (+)	10.39 ± 8.54	13.4 ± 8.98	0.007

*p* value^†^	0.402		

^*∗*^2004 versus 2007 IPSS in each group (paired *t*-test).

^†^2004 versus 2007 IPSS between MS (−) and MS (+) (2-way ANOVA test).
